# Selective Arterial Embolization of Liver Metastases from Gastrinomas: A Single-Centre Experience

**DOI:** 10.1155/2013/174608

**Published:** 2013-07-29

**Authors:** Anneke P. J. Jilesen, Heinz Josef Klümpen, Olivier R. C. Busch, T. M. van Gulik, Krijn P. van Lienden, Dirk J. Gouma, Els J. M. Nieveen van Dijkum

**Affiliations:** ^1^Department of Surgery, Academic Medical Center, Meibergdreef 9, P.O. Box 22660, 1105 AZ Amsterdam, The Netherlands; ^2^Department of Medical Oncology, Academic Medical Center, Meibergdreef 9, P.O. Box 22660, 1105 AZ Amsterdam, The Netherlands; ^3^Department of Intervention Radiology, Academic Medical Center, Meibergdreef 9, P.O. Box 22660, 1105 AZ Amsterdam, The Netherlands

## Abstract

*Background*. Gastrinomas are rare functional neuroendocrine tumors causing the Zollinger-Ellison syndrome (ZES). At presentation, up to 25% of gastrinomas are metastasized, predominantly to the liver. Embolization of liver metastases might reduce symptoms of ZES although a postembolization syndrome can occur. In this study, the results of embolization are presented, and the literature results are described. *Methods*. From a prospective database of pancreatic neuroendocrine tumors, all patients with liver metastatic gastrinomas were selected if treated with arterial embolization. Primary outcome parameters were symptom reduction, complications, and response rate. The literature search was performed with these items. *Results*. Three patients were identified; two presented with synchronous liver metastases. All the three patients had symptoms of ZES before embolization. Postembolization syndrome occurred in two patients. Six months after embolization, all the 3 patients had a clinical and complete radiological response; a biochemical response was seen in 2/3 patients. From the literature, only a small number of gastrinoma patients treated with liver embolization for liver metastases were found, and similar results were described. *Conclusion*. Selective liver embolization is an effective and safe therapy for the treatment of liver metastatic gastrinomas in the reduction of ZES. Individual treatment strategies must be made for the optimal success rate.

## 1. Introduction

Gastrinomas are neuroendocrine tumors (NET), primarily located in the duodenum or pancreas. Gastrinomas are by definition functional tumors secreting gastrin. Gastrin overproduction causes the Zollinger-Ellison syndrome, which includes ulceration of the gastrointestinal tract, mainly the jejunum, resulting in abdominal pain and diarrhea [1]. The incidence of gastrinomas is 0.5–2 per million per year and therefore very rare [2, 3]. Gastrinomas are classified according to a grading system, similar to other pancreatic neuroendocrine tumors (pNETs). This grading is based on histopathology and subdivided into immunostaining for tumor markers and proliferation markers (Table 1) [4]. Using the current WHO criteria, grades 1 and 2 are well-differentiated pNETs with increased expression of the tumor markers, chromogranin A, and synaptophysin. Grade 3 tumors are poorly differentiated with areas of necrosis and decreased expression of chromogranin A [3, 5].

Up to 25% of the gastrinomas are diagnosed when metastases are already present, predominantly in the liver. Liver metastases are the most important prognostic factor for survival [2, 6]. Ten-year survival of patients with diffuse liver metastases is 16% compared to 90% 10-year survival in patients who underwent a curative gastrinoma resection [2]. For patients with unresectable liver metastases, hepatic artery embolization (TAE) is a therapeutic option to reduce metastatic symptoms. Patients with liver metastases may experience symptoms such as weight loss, pain, and anorexia, particularly caused by tumor load. Liver metastases derive the majority of their blood supply from the hepatic artery, compared with normal liver parenchyma, which derive the majority of the blood supply from the portal venous circulation. Embolization results in tumor reduction and therefore symptom reduction [7]. Postembolization syndrome is the most important complication after embolization, characterized by symptoms of fever, unremitting nausea, general malaise, loss of appetite, and abdominal pain. The exact cause is not yet entirely clarified; however, it may be a result of tumor ischemia and inflammation of the liver tissue [8, 9].

Only a small series describes the effect of hepatic embolization of liver metastases from gastrinomas. The aim of this study is to present our single-centre experience of the effect of selective arterial embolization for gastrinomas in symptoms reduction, complications, and response rate. These results are compared to the literature results, and a protocol for patients care during embolization is presented. 

## 2. Patients and Methods

All patients with liver metastatic gastrinomas, treated by selective hepatic artery embolization, were selected from a prospective database starting in January 1992 up till December 2012. 

Data concerning clinical presentation, previous treatment, and embolization treatment were studied. Diagnostic strategy for gastrinoma patients includes serum chromogranin A and gastrin levels, preferably after a 10-day cessation of the proton pump inhibitors (PPIs). Imaging is then performed with CT scan, Octreoscan, and sometimes EUS. 

Our treatment protocol for gastrinoma patients consists of a resection in patients with a solitary resectable primary lesion or a resectable primary lesion with resectable liver metastases. Patients with a gastrinoma and irresectable liver metastases do not undergo resection of the primary gastrinoma. 

Patients with irresectable liver metastases are treated with PPI's sometimes combined with somatostatin analogues. The indication for embolization is an insufficient response to medical treatment for relief of symptoms or progressive disease confined to the liver. If embolization is not possible or patients have progressive disease after embolization therapy, further chemotherapeutical options or peptide receptor radionuclide therapy options are discussed. 

**Figure 1:**
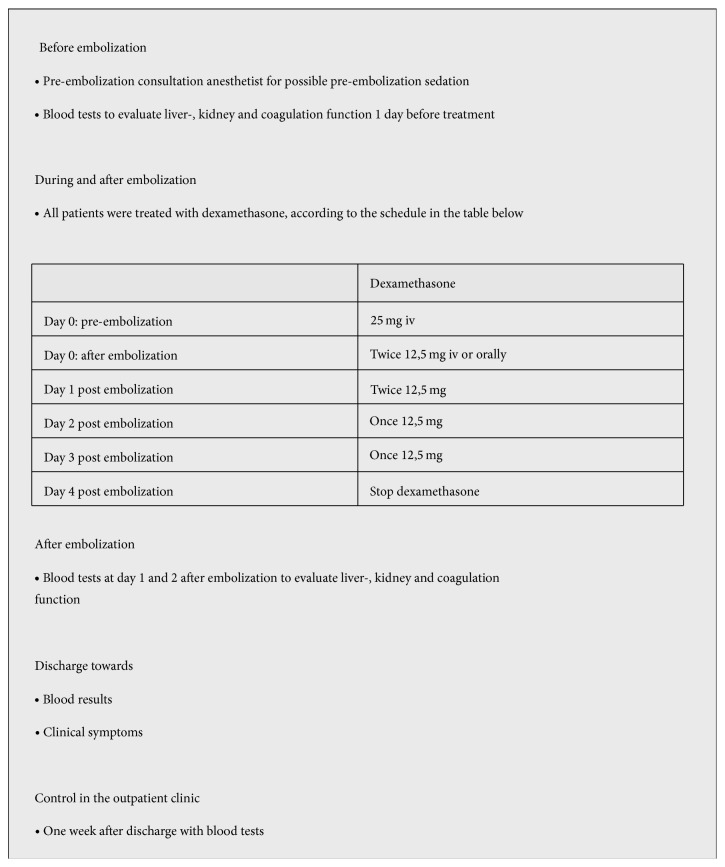
Local embolization protocol for noncarcinoid liver metastases [10].

All patients were treated according a local embolization protocol (Figure 1) [10]. Complication rate and the effect of embolization were examined. Embolization response is evaluated according the Response Evaluation Criteria In Solid Tumors (RECIST) [11]. Patients were considered in complete response (CR) if gastrin or chromogranin levels were normal and target lesions disappeared. A partial response (PR) was considered if at least 30% reduction was achieved of the tumor markers or target lesions. The progression of disease (PD) is described as ≤20% increase of tumor makers or if new lesions were noticed. Time to followup is still ongoing or ended due to death of the patients. All information was collected from hospital medical records. 

## 3. The Literature Search

The literature search was performed in PubMed and Embase using the following headings: gastrinoma, liver metastases, neuroendocrine tumors, carcinoid, Zollinger-Ellison syndrome, and embolization. In the included studies, the references were searched for other manuscripts. All abstracts in English which evaluate hepatic artery embolization for liver metastases in patients with NET were included. Studies were excluded if patients were treated with hepatic arterial chemoembolization (TACE) alone instead of hepatic artery embolization and if treated patients had no gastrinoma or pNET, such as midgut carcinoid or lung NET. 

## 4. Results of the Patient Study

### 4.1. Clinical Presentation

Three patients were identified (Table 2) from a total of 109 pNET patients in the database including 13 gastrinomas. Of the 13 gastrinoma patients, 1 patient had lymph nodes metastases and will not be further discussed; 11 patients were previously described [12]. Three patients had liver metastasis and were included in this study. 

Patient 1 is a 60-year-old female without comorbidity. She had symptoms of reflux disease, abdominal pain, and diarrhea for years. After an episode of undesirable weight loss of 15 kg, an ultrasound showed single liver metastases. No extrahepatic localization of gastrinoma was found.

Patient 2 is a 43-year-old male without other morbidities. At presentation, there was a disease-free survival of 76 months after resection of a pancreatic gastrinoma. Then symptoms of the Zollinger-Ellison syndrome recurred caused by resectable liver metastases. After a 6/7 liver segment resection, symptoms returned 16 months later because of new liver metastases. 

Patient 3 is a 58-year-old man with a previous history of diverticulitis which was treated with a sigmoid resection. He also suffered from reflux complaints for years. He presented with a duodenal perforation and underwent a damage control laparotomy and primary closure of the defect with a prolonged hospital stay including multiple drainage procedures and intensive care episodes. Imaging during this episode also showed multiple right-sided liver metastases and a gastrinoma of the pancreas.

### 4.2. Pre-Embolization

All patients had symptoms of the Zollinger-Ellison syndrome. Symptoms included diarrhea, abdominal pain, and heartburn. Symptoms were treated with a PPI (40 mg twice a day), and patients had increased symptoms when these PPIs were discontinued, for example, for blood tests for chromogranin A and gastrin measurements. All patients had elevated levels of chromogranin A and/or gastrin (Table 3) before embolization. 

Indications for embolization were patients' choice of treatment for embolization instead of liver resection in patient 1 and multiple liver metastases in left and right liver in patient 2. The third patient had major complications after duodenal perforation combined with Zollinger-Ellison syndrome, and embolization was performed to reduce symptoms while recovering before the curative gastrinoma and right liver resection could be performed. 

### 4.3. Embolization

**Figure 2:**
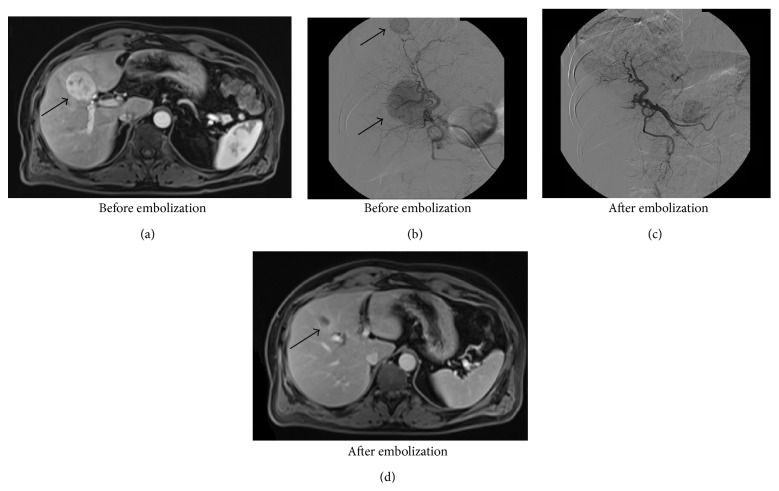
Imaging before and after embolization. Before embolization; in (a), a central lesion (pointed by the arrow) is shown on MRI, and in (b), two spherical abnormalities (pointed by the arrows) are shown on angiography, caused by hypervascularization of two metastases. After successful embolization; in (c), only normal liver parenchyma is remaining, and after 6 months, only a small necrotic lesion is left (pointed by the arrow) in (d) on MRI.

All patients were punctured in the right artery femoralis. Selective embolization was performed with polyvinyl alcohol (PVA) particles of the selective left or right hepatic artery (Figure 2). In these three patients, embolization was performed as a one-stage procedure, regardless if the lesions were diffusely spread in the left and right liver. In patient 1, extreme selective embolization of the right hepatic artery occurred, in patient 2, both arteries, and in patient 3, only the right hepatic artery. Patient 1 started selective embolization treatment in 2010, patient 2 in 2007, and patient 3 in 2010. 

### 4.4. Post-Embolization

Patients 2 and 3 developed a postembolization syndrome (Table 3). Patient 2 developed symptoms of fever, general malaise, and abdominal pain 4 days after embolization. A CT scan showed necrosis of the tumor. The patient was readmitted for observation without the need for a reintervention or antibiotics. Patient 3 experienced symptoms of general malaise, chest pain, nausea, and sweating without a cardiac or pulmonary cause. The patient was observed for one day extra without any further complications or reinterventions. 

All patients experienced a clinical response; in patients 2 and 3, the PPIs dose was reduced by 50% without worsening of symptoms. In all patients, symptoms of diarrhea or abdominal pain completely disappeared after embolization. Chromogranin A and gastrin levels declined after embolization in two patients (Table 3). Patient 2 had diffuse disease progression of other new liver lesions, including rise of gastrin level. No liver or renal function impairment occurred.

All patients had a complete radiological response (Table 3) after 6 months of the treated liver lesions. Patient 2, with diffuse liver metastasis, had tumor progression of new liver metastases. After embolization, he also underwent radiofrequency ablation (RFA) therapy, surgical metastasectomy, and finally peptide receptor radionuclide therapy (PRRNT). He died 162 months after the initial pancreatic resection and 41 months after embolization therapy due to progressive disease including pancytopenia and aplastic anemia after recent PPRNT. Patient 3, with diffuse liver metastasis in the right liver lobe, was eligible for surgical resection of pancreatic gastrinoma and liver metastases after embolization which successfully took place, one year after embolization. 

Progression-free survival (PFS) of patient 1 is 20 months after embolization. Because of recurrent increase in gastrin and chromogranin A, a second embolization was performed 22 months after the first embolization. In patient 2-no progression-free survival can be related to embolization because of the diffuse progression of new liver metastases. Patient 3 has a PFS of 26 months after embolization and is currently still tumor free but also underwent a right hemihepatectomy 12 months after embolization. 

## 5. Results of the Literature Research

An overview of the literature research is shown in Table 4. Less is known about the effect of liver embolization for liver metastases in patients with pNET, given the amount of publications. Therefore, all studies regarding pNET and liver metastases treated with liver embolization are listed in Table 4. A total of 13 studies seemed suitable for analysis. A further 9 studies included only carcinoid patients with liver metastases and were excluded. All studies were unclear in presenting the results for gastrinomas separately. Of all the studies, 8 studies only described the results of pNET without specifying the presence of gastrinomas [13–20]. Some of the studies mentioned the number of gastrinoma patients but not the specific individual response. It is not described if the presented results apply for the included gastrinoma patients. 

Two studies have included patients treated with TAE or TACE [15, 21] without differentiation by types of treatment. Five studies have included gastrinomas [21–25] all presented an objective response after embolization for the total group of included patients. A clinical response is seen, from symptom relief up to a complete response in 59% of the patients, [22–24] and a biochemical response is described with a reduction of hormone levels in 50% [23–25]. The radiological response is not uniformly described, from the presence of tumor regression to a partial response of 67% [21, 24, 25]. 

## 6. Discussion

All patients with liver metastatic gastrinoma in the present study treated with selective hepatic artery embolization showed a clinical, biochemical, and radiological response. Although this is only a small series, some lessons can be learned. 

Selective embolization of liver metastases resulted in symptom reduction in all patients. Before embolization, patients suffer from the typical Zollinger-Ellison syndrome, which impaired their daily life due to symptoms of the Zollinger-Ellison syndrome. After selective embolization, besides heartburn, treated with only half doses of PPI, there were no symptoms left. Decreased chromogranin A and gastrin levels were achieved after embolization (Table 3). A complete radiological response was seen in all patients in the treated lesions after 6 months. Therefore, selective embolization of gastrinoma liver metastasis seems to be an effective treatment for the reduction of symptoms. This is also shown in other studies including different pNET and carcinoid patients. 

In general, surgical treatment is the first curative option [12] in patients with a gastrinoma. Liver embolization is only effective on treated lesions, other metastases remain untreated. In patients with tumors that demonstrate aggressive growth, an additional treatment is recommended [26]. In metastatic gastrinomas, surgical resection remains the best option for extended survival [27]. In patients with diffuse liver metastases, surgical resection is often technically impossible. Different kinds of treatment options, including RFA, radioembolization, and PRRT, are available. Major complications of these treatments can occur. The toxic effects after RFA treatment may lead to liver abscesses, cholangitis, hepatic infarction, subcutaneous abscesses, and pleural effusion [28]. Radioembolization may lead to pancytopenia caused by bone marrow suppression, pulmonary insufficiency secondary to radiation pneumonitis, gastric and duodenal ulceration, and ascites [29]. PRRT can cause renal insufficiency, myelodysplastic syndrome, and leukaemia [30]. Compared to these treatments, toxicity of TAE is relatively mild [8] as was also shown in our patients. 

Patients were treated according to our own institute embolization protocol to prevent the postembolization syndrome which is described in 82% of patients [8]. Nevertheless, two out of three patients suffered from mild symptoms of postembolization syndrome resulting in extended hospital stay of one day and readmission of 2 days. Since liver abscess is very infrequent in patients without previous bile duct involving operations and described to be only 0,4%, no standard antibiotic prophylaxes or treatment are added to the protocol. However, the patient with previous pancreatic and liver resections was treated with antibiotics during the hepatic artery embolization to prevent infectious complications. This NET embolization protocol does include treatment with corticosteroids, as described in patients with HCC. The rationale for corticosteroids is not only edema prevention for decreased liver capsular pressure and subsequent pain reduction but also to reduce the immune response [31, 32]. 

Only five studies describe detailed results of hepatic artery embolization in gastrinoma patients (Table 4). Most studies show results of embolization in pNET and carcinoid patients without differentiation for tumor type or tumor grade/stage. Overall results of hepatic artery embolization of patients with pNET liver metastases show a 17–100% effect on either clinical, biochemical, or radiological measurements. Complications and postembolization syndrome were not well described in these studies and could not be compared to our results. This is the first study describing the exact response rate of TAE in liver metastatic gastrinomas. 

## 7. Conclusion 

Gastrinoma with liver metastases is a rare condition with a broad spectrum of treatment strategies [2, 3]. Selective hepatic artery embolization leads to symptom control of the Zollinger-Ellison syndrome in liver metastatic gastrinomas. Post embolization syndrome is the most important complication after hepatic artery embolization; therefore, careful treatment protocols must be available for prevention of tumor necrosis syndrome after posthepatic artery embolization. Our advice is to discuss every patient in a multidisciplinary meeting with endocrinologists, oncologists, pathologists, HPB surgeons, and (intervention) radiologists. Treatment should be based on their tumor classification, tumor burden, complaints, and quality of life considerations. 

## Figures and Tables

**Table 1:** Tumor grade of gastrinomas based on proliferation markers [4].

Tumor grade	Mitotic count	Ki67 index
G1	1	≤2
G2	2–20	3–20
G3	>20	>20

**Table 2:** Clinical characteristics of patients undergoing embolization for liver metastases of gastrinomas.

Patient	Gender	Age (years)	Clinical presentation	Tumor classification	Previous treatment	Location of liver metastases	Number of lesions	Diameter (cm.)
1	F	60	Reflux, diarrhea, and weight reduction	Well-differentiated NET, G1	None	Segment 6	1	6.2 × 3.6 cm.

2	M	43	Reflux, diarrhea	Well-differentiated NET, G2	Resection liver metastases segment 6-7	Segment 2-3-4-5-8	6	Segment 2: 2.4 cm. Segment 3: 4.0 cm. Segment 4: 3.4 cm. Segment 5: 2.1 cm. Segment 8: 2.7 and 1.9 cm.

3	M	58	Duodenal perforation	Well-differentiated NET, G1	Long acting somatostatin analogues	Segment 5-7-8	3	Segment 7/8: 2.2 cm. Segment 5: 4.0 and 2.6 cm.

**Table 3:** Response rate and blood results after embolization of treated liver lesions.

Patient	Complication rate	Biochemical response at 6 months	Gastrin level before → after embolization	Chromogranin level before → after embolization	Radiological response after 6 months
1	No	CR	2300 → 50	4400 → 232	CR
2	Postembolization syndrome	PD∗	345 → 1625∗	NS	CR
3	Postembolization syndrome	CR	500 → 65	2160 → 96	CR segment 7/8 PR both in segment 5

^*^Patient 2 had progressive disease with new liver metastasis and therefore additional treatment within 6 months after embolization was required. An explicit biological response of the embolization treatment was not possible because of the increased levels of gastrin due the new lesions. No chromogranine A was determined for patient 2.

**Table 4:** The literature research.

Author Year	*N *	Location of primary tumor	Number of gastrinomas	Treatment	Clinical response (overall) *N* (%)∗	Biochemical response *N* (%)∗	Radiological response∗
Sato et al., 2000 [13]	2	pNET	NS	TAE	NS	100%	NS

Mitty et al., 1985 [14]	18	Carcinoid, pNET, lung, unknown	NS	TAE	17 (94%)	12 (67%)	NS

Kamat et al., 2008 [15]	38	pNET, carcinoid	NS	TAE TACE	20 (53%)	NS	15 (44%)

Strosberg et al., 2006 [16]	84	Carcinoid, unknown, lung	NS	TAE	44 (52%)	35 (42%)	40 (48%)

Meij et al., 2005 [17]	13	pNET, carcinoid	NS	TAE	4 (31%)	3 (23%)	4 (31%)

Chamberlain et al., 2000 [18]	33	pNET, carcinoid	NS	TAE	31 (94%)	NS	NS

Marlink et al., 1990 [19]	10	pNET, carcinoid	NS	TAE	10 (100%)	10 (100%)	10 (100%)

Stockmann et al., 1984 [20]	6	pNET, carcinoid	NS	TAE	6 (100%)	6 (100%)	NS

Gupta et al., 2005 [21]	123	pNET, carcinoid, lung, unknown	9	TAE TACE	NS	NS	67% carcinoid 35% pNET

Osborne et al., 2006 [22]	59	Carcinoid, pNET, unknown, lung	2	TAE	48 (81%)	NS	NS

Ajani et al., 1988 [23]	22	pNET	9	TAE	12 (55%)	12 (55%)	NS

Brown et al., 1999 [24]	35	pNET, carcinoid	4	TAE	10 (29%)	12 (34%)	12 (34%)

Eriksson et al., 1998 [25]	41	pNET, carcinoid	2	TAE	NS	16 (40%)	38% carcinoid 17% pNET

Jilesen, present study	3	Gastrinomas	3	TAE	3 (100%)	2 (67)	3 (100%)

^*^None of the studies described the response rate for gastrinomas separately. The results presented in the table describe the overall response rate of embolization in liver metastases from pNET/carcinoid.
